# Basic fibroblast growth factor‐loaded methacrylate gelatin hydrogel microspheres for spinal nerve regeneration

**DOI:** 10.1002/SMMD.20220038

**Published:** 2023-03-28

**Authors:** Xiaoyan Chen, Lei Ren, Hui Zhang, Yangnan Hu, Menghui Liao, Yingbo Shen, Kaichen Wang, Jiaying Cai, Hong Cheng, Jiamin Guo, Yanru Qi, Hao Wei, Xiaokun Li, Luoran Shang, Jian Xiao, Jingwu Sun, Renjie Chai

**Affiliations:** ^1^ State Key Laboratory of Bioelectronics Department of Otolaryngology Head and Neck Surgery Zhongda Hospital School of Life Sciences and Technology Advanced Institute for Life and Health Jiangsu Province High Tech Key Laboratory for Bio‐Medical Research Southeast University Nanjing China; ^2^ Chien‐Shiung Wu College Southeast University Nanjing China; ^3^ Department of Otolaryngology Head and Neck Surgery Affiliated Drum Tower Hospital of Nanjing University Medical School Nanjing China; ^4^ Co‐Innovation Center of Neuroregeneration Nantong University Nantong China; ^5^ School of Pharmaceutical Sciences Wenzhou Medical University Wenzhou Zhejiang China; ^6^ Shanghai Xuhui Central Hospital Zhongshan‐Xuhui Hospital The Shanghai Key Laboratory of Medical Epigenetics, the International Co‐laboratory of Medical Epigenetics and Metabolism (Ministry of Science and Technology) Institutes of Biomedical Sciences Fudan University Shanghai China; ^7^ Oujiang Laboratory (Zhejiang Lab for Regenerative Medicine, Vision and Brain Health) Wenzhou Zhejiang China; ^8^ Department of Otolaryngology‐Head and Neck Surgery The First Affiliated Hospital of USTC Division of Life Sciences and Medicine University of Science and Technology of China Hefei Anhui China; ^9^ Department of Otolaryngology Head and Neck Surgery Sichuan Provincial People's Hospital University of Electronic Science and Technology of China Chengdu China; ^10^ Institute for Stem Cell and Regeneration Chinese Academy of Sciences Beijing China; ^11^ Beijing Key Laboratory of Neural Regeneration and Repair Capital Medical University Beijing China

**Keywords:** drug delivery, GelMA microsphere, neurotrophic factors, spinal cord injury, tissue engineering

## Abstract

Spinal cord injury is a severe central nervous system injury, and developing appropriate drug delivery platforms for spinal nerve regeneration is highly anticipated. Here, we propose a basic fibroblast growth factor (bFGF)‐loaded methacrylate gelatin (GelMA) hydrogel microsphere with ideal performances for spinal cord injury repair. Benefitting from the precise droplet manipulation capability of the microfluidic technology, the GelMA microspheres possess uniform and satisfactory size and good stability. More importantly, by taking advantage of the porous structures and facile chemical modification of the GelMA microspheres, bFGF could be easily loaded and gradually released. By co‐culturing with neural stem cells, it is validated that the bFGF‐loaded GelMA microspheres could effectively promote the proliferation and differentiation of neural stem cells. We also confirm the effective role of the bFGF‐loaded GelMA microspheres in nerve repair of spinal cord injury in rats. Our results demonstrate the potential value of the microspheres for applications in repairing central nervous system injuries.

## Graphical abstract

The carboxylated methacrylate gelatin (GelMA) microspheres were combined with the amino group of basic fibroblast growth factor (bFGF) to obtain bFGF‐GelMA microspheres. bFGF‐GelMA microspheres were injected into the site of spinal cord injury, and bFGF was released slowly. Endogenous neural stem cells continue to proliferate and differentiate under the stimulation of bFGF, promoting the regeneration of damaged nerves.

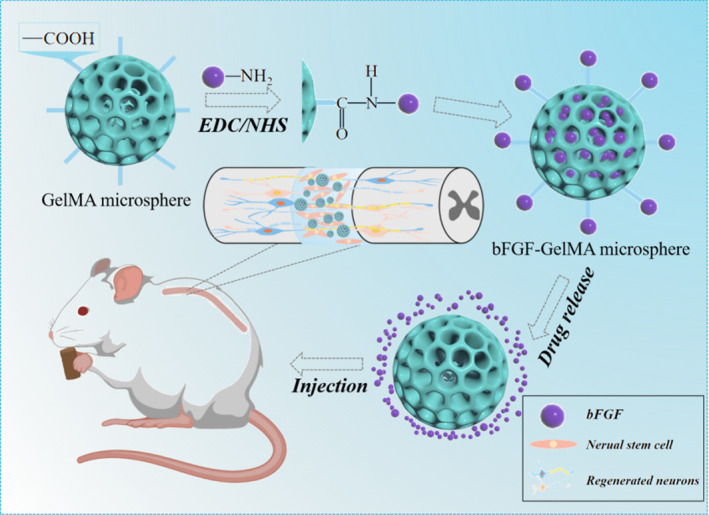

1

**Key points**

- The preparation method of basic fibroblast growth factor (bFGF)‐loaded methacrylate gelatin (GelMA) microspheres was described.
- A platform for efficient targeted delivery of bioactive molecule bFGF was designed.
- The application of bFGF‐loaded GelMA microspheres in the repair of spinal cord injury was introduced.

## 2 INTRODUCTION

Spinal cord injury (SCI) is one of the traumatic injuries of the central nervous system, which often results in severe dysfunction of the limbs and organs below the injured segment.^1^, ^2^, ^3^ Tissue‐engineered constructs and scaffolds have attracted extensive attention for nerve repair by supplying a physical support and delivering therapeutic agents, such as neurotrophic and angiogenic factors, as well as stem cells to promote blood vessel reconstruction,^4^, ^5^ axon growth,^6^, ^7^ and nerve regeneration.^8^, ^9^, ^10^ However, current tissue scaffolds are typically bulk materials, whose lack of structural flexibility might restrict their final performances.^11^, ^12^, ^13^ Alternatively, microspheres have emerged as an efficient platform for the encapsulation and targeted delivery of bioactive factors.^14^, ^15^, ^16^ Microsphere‐based delivery systems can effectively protect encapsulated factors from degradation and help maintain a relatively high concentration at the target area.^17^, ^18^, ^19^ Although significant achievements have been made, a large portion of them face the dilemma of the complicated material synthesis process, nonuniform particle size, or inefficient drug loading.^20^, ^21^ These drawbacks limit their widespread applications in nerve regeneration; hence, a novel microsphere delivery platform for spinal nerve regeneration is highly anticipated.

**FIGURE 1:**
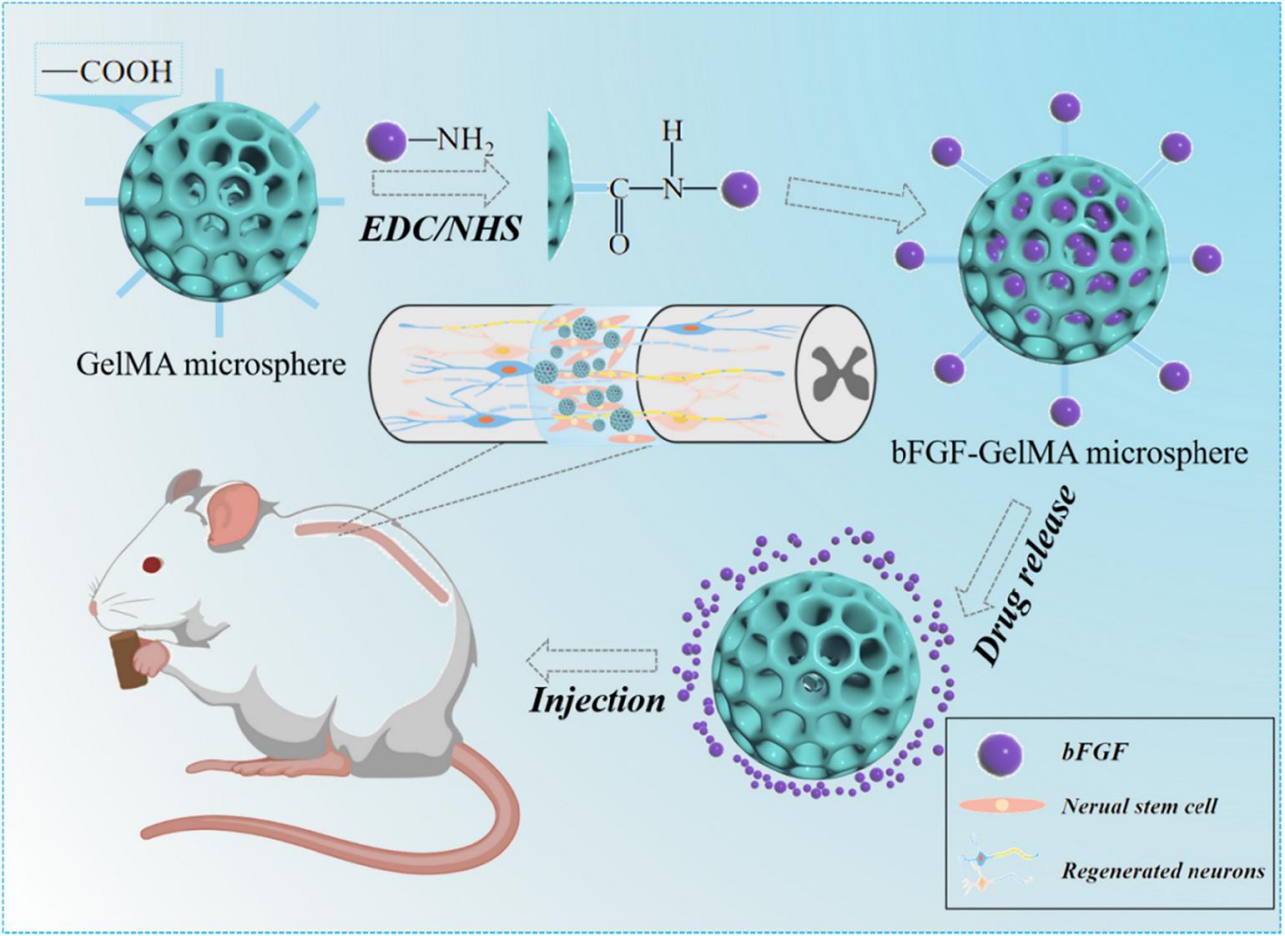
Schematic diagram of basic fibroblast growth factor‐loaded methacrylate gelatin (bFGF‐GelMA) hydrogel microspheres for spinal cord injury repair. The GelMA microspheres treated with N‐(3‐Dimethylaminopropyl)‐N’‐ethylcarbodiimide hydrochloride (EDC)/N‐Hydroxy succinimide (NHS) have activated carboxyl groups and combine with the amino groups of bFGF to obtain bFGF‐GelMA microspheres. Then, the bFGF‐GelMA microspheres were injected into the site of spinal cord injury, and the bFGF was released slowly. Endogenous neural stem cells continue to proliferate and differentiate under the stimulation of bFGF to promote the regeneration of injured nerves.

In this paper, we proposed microfluidic‐derived hydrogel microspheres encapsulating basic fibroblast growth factor (bFGF) for spinal cord injury repair as shown in Figure 1. fFurthermore, the small size of the hydrogel microspheres allows them to be injected and inhaled through small needles and catheters, facilitating minimally invasive delivery of cells and biologicals. In addition, hydrogel microspheres have significant porosity. The level of the porosity is related to the size and packing density of hydrogel microspheres, which can regulate the proliferation and migration of supporting cells and effectively control the release of biological agents for long‐term therapeutic effects.^28^ On the other hand, bFGFs refer to a family of growth factors that participate in the modulation of angiogenesis in various pathological conditions.^29^ Prominently, evidence has confirmed the therapeutic role of bFGFs in promoting spinal cord repair.^30^, ^31^ Therefore, it was conceived that by combining the merits of the microfluidic technology and bFGF, novel microspheres could be fabricated for bFGF delivery and SCI treatment.

Herein, we utilized microfluidic technology to prepare bFGF‐loaded methacrylate gelatin (GelMA) hydrogel microspheres. Microfluidic emulsion droplets of GelMA pre‐gel were formed through a capillary microfluidic device and polymerized in situ by ultraviolet (UV) irradiation. The resultant microspheres exhibited a uniform size as well as excellent biocompatibility. Besides, the GelMA microspheres could be endowed with an interconnected porous structure after freeze‐drying, which facilitated efficient loading of bFGF. We demonstrated through in vitro experiments that the bFGF‐loaded GelMA microspheres significantly promoted the proliferation and differentiation of neural stem cells. Moreover, we confirmed the role of the bFGF‐loaded GelMA microspheres in spinal nerve regeneration in vivo through a rat model. Benefiting the efficient loading and sustained release capacities of the microspheres, bFGF could maintain an effective concentration and prolonged action time in the spinal cord injured area, thereby promoting neuronal differentiation and motor function reconstruction. These results strongly supported that the present bFGF‐loaded GelMA microspheres have great potential in drug delivery and SCI recovery.

## 3 RESULTS AND DISCUSSION

### 3.1 Preparation and characterization of GelMA microspheres

**FIGURE 2:**
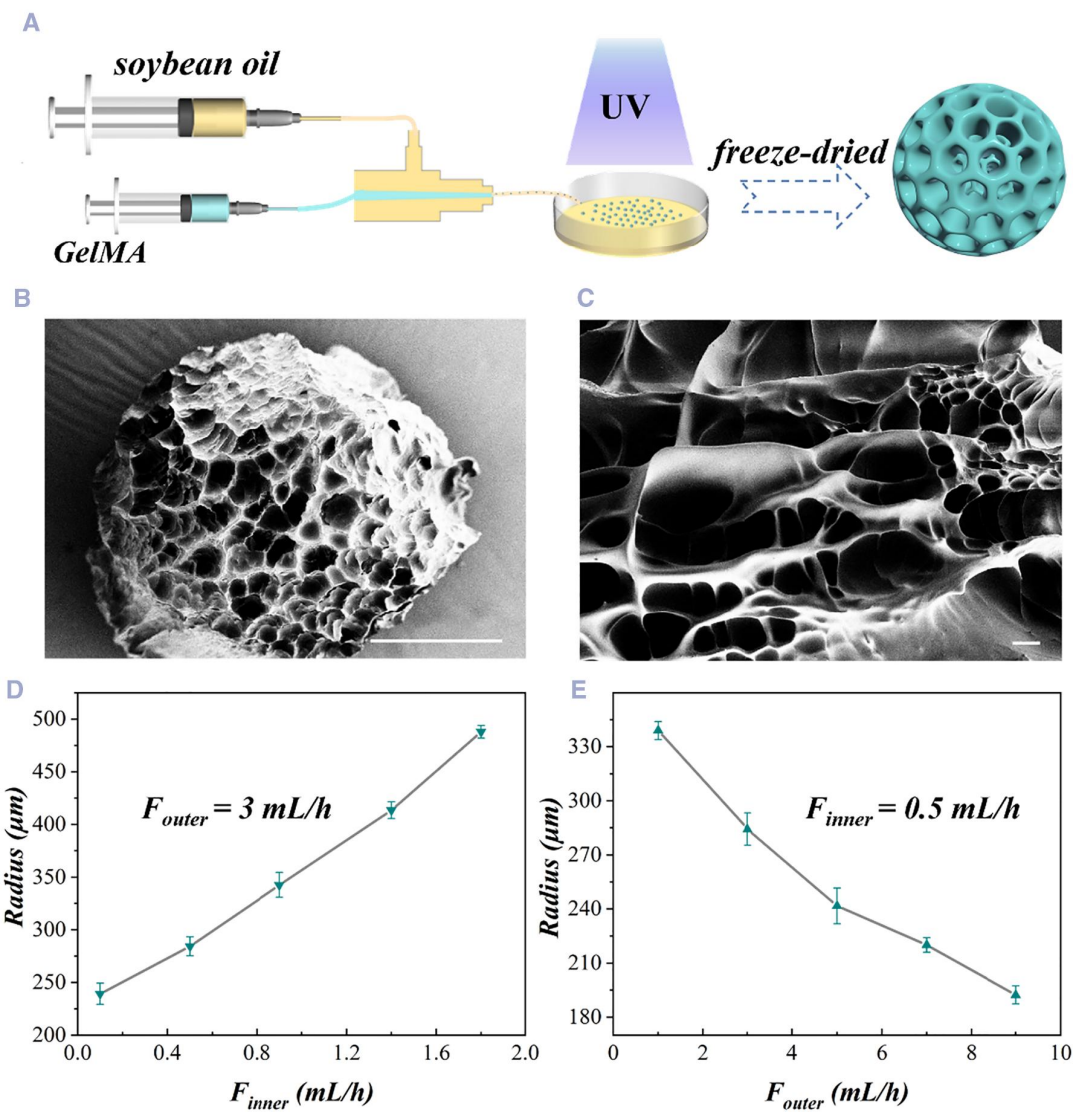
Preparation of methacrylate gelatin (GelMA) microspheres. (A) Schematic diagram of the preparation process of the GelMA microspheres. (B) Scanning electron microscope (SEM) images of the GelMA microsphere and (C) the surface morphology. (D) The relationship between the diameter of the microsphere and the inner flow rate. The flow rate of the outer phase was set at 3 mL/h. (E) The diameter of the microspheres as a function of the outer flow rate. The inner flow rate was set at 0.5 mL/h. Scale bars are 200 μm in (B) and 20 μm in (C).

In a typical experiment, GelMA microspheres were generated by using microfluidic technology. Single emulsion droplets were first formed through a microfluidic device consisting of two cylindrical capillaries arranged coaxially in a square capillary. The inner phase fluid and outer phase fluid of incompatible flowed through the injection tube and the collection tube, respectively, and formed a two‐phase co‐flow system. The inner phase was a mixture of GelMA pregel solution and photoinitiator, and the outer phase was soybean oil. Monodisperse droplets were generated continuously in the collection tube of the device under the action of shear force and interfacial tension between the two phases. The droplets were then cross‐linked in situ under UV irradiation, after which they were washed with absolute ethyl alcohol, thus generating GelMA hydrogel microspheres as shown in Figure 2A. The microstructure of the resultant hydrogel microspheres after lyophilization was characterized by a scanning electron microscope (SEM), which showed that the freeze‐dried microspheres were porous (Figure 2B,C). Besides, the overall size of the GelMA microspheres could be controlled by adjusting the flow rates of the inner and outer phases. In the case of a fixed inner phase flow rate, the size of droplets and the microspheres decreased with the increase of the phase flow rate (Figures 2D and S1A). When the flow rate of the outer phase was fixed, the size of microspheres increased with the inner phase flow rate (Figure 2E). In all cases, the as‐prepared GelMA hydrogel microspheres showed the uniform spherical shape and narrow size distribution (Figure S1B).

**FIGURE 3:**
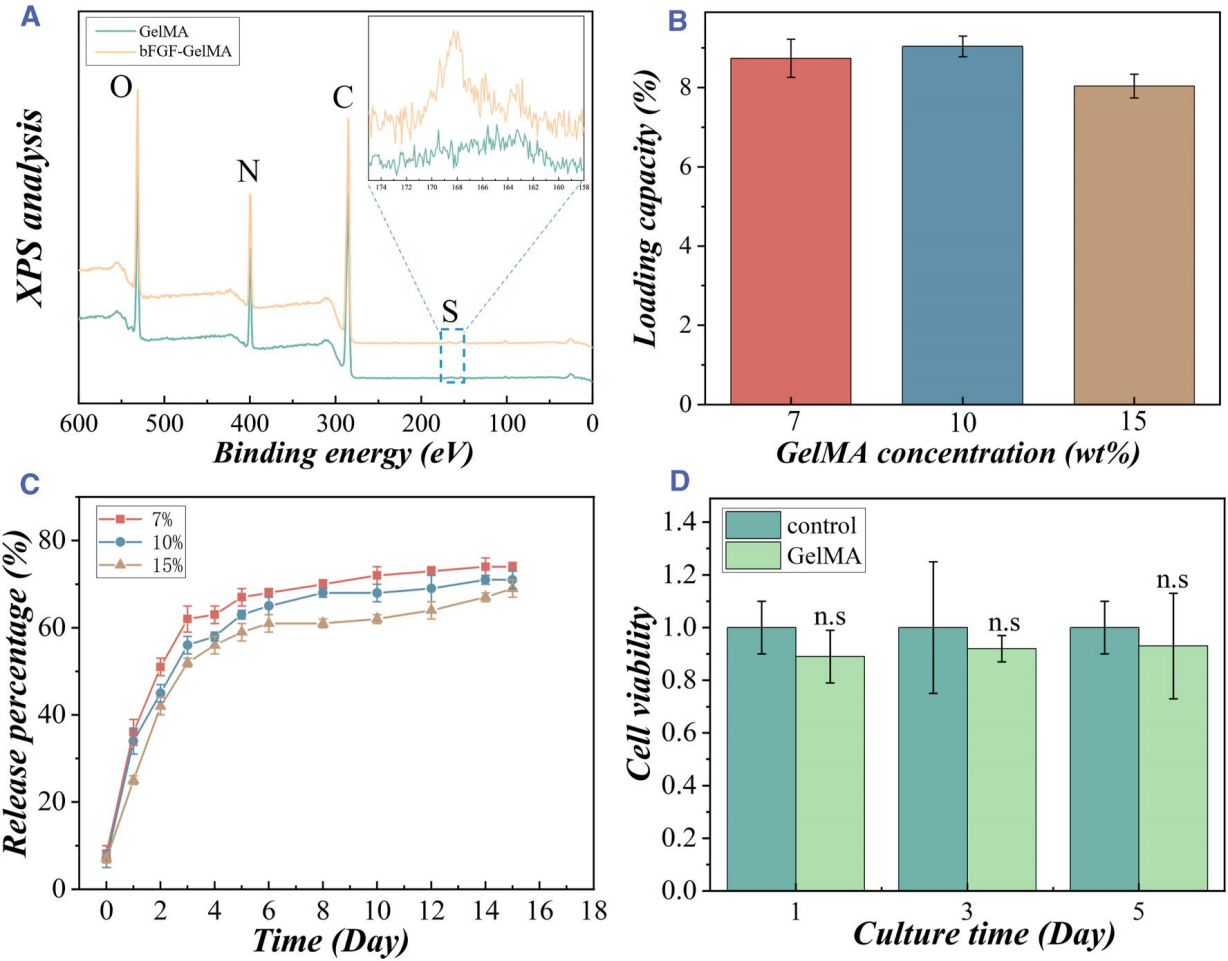
Characterization of the basic fibroblast growth factor‐loaded methacrylate gelatin (bFGF‐GelMA) microspheres. (A) X‐ray photoelectron spectroscopy (XPS) analysis of the GelMA hydrogel microspheres and bFGF‐loaded GelMA microspheres. (B) The drug loading capacity of the GelMA hydrogel microspheres with different GelMA concentrations. (C) Drug release profile of the GelMA hydrogel microspheres. (D) Effect of the GelMA microspheres on the biological activity of NSCs. Experiments were repeated five times, and the data were normalized to the control group cell viability each day. Error bars represent the standard deviation.

bFGF was loaded in the GelMA microspheres by soaking the microspheres in a bFGF solution. In detail, the freeze‐dried hydrogel microspheres were treated with EDC and NHS to activate the carboxyl groups. After this, the microspheres were covalently bonded with the amino residues of bFGF to form bFGF‐loaded GelMA hydrogel microspheres. Next, the X‐ray photoelectron spectroscopy (XPS) analysis of the GelMA microspheres and bFGF‐loaded GelMA microspheres was conducted. As shown in Figure 3A, the peak values of carbon (C 284.8 eV), nitrogen (N 400.0 eV), and oxygen (O 532.0 eV) had no difference between the GelMA microsphere and the bFGF‐loaded GelMA microsphere. By contrast, there was no sulfur peak (S 167.8 eV) in the GelMA microspheres, whereas the bFGF‐GelMA microspheres had a sulfur peak. The S element was attributed to the disulfide bond in bFGF, which indicated that bFGF had been successfully integrated into the GelMA microspheres. In addition, Fluorescein Isothiocyanate‐bovine serum albumin (FITC‐BSA) is a common model drug. The total amount of drug release can be calculated by detecting the absorbance or fluorescence intensity of the fluorescein isothiocyanate (FITC, a fluorescent molecule). Based on this, we studied the drug loading capacity of GelMA hydrogel using FITC‐BSA as a model drug. Specifically, microspheres with different GelMA concentrations were treated with EDC/NHS and then combined with FITC‐BSA. As shown in Figure 3B, the drug loading capacity did not change significantly with the hydrogel concentration.

Thereafter, we measured the release of FITC‐BSA from the GelMA microspheres in the collagenase environment using a microplate reader. The release curves of the GelMA microspheres with different hydrogel concentrations over 15 days were recorded as shown in Figure 3C. As expected, the drug release rate was initially rapid within the first 3 days and then became relatively slow over the following days. It was also found that the microspheres with a lower hydrogel concentration released relatively faster than those with a higher hydrogel concentration. As seen in the subsequent enzymatic degradation experiments, it was speculated that this may be attributed to the slower overall degradation of the hydrogel at high concentrations, resulting in a relatively slower rate of drug release. Besides, to simulate the in vivo environment, we recorded the degradation rate of GelMA microspheres with different hydrogel concentrations in a 2 μg/mL collagenase solution. As shown in Figure 2, after 25 days, the microspheres reached 100% degradation, and the microspheres with a higher hydrogel concentration showed slower degradation. This might be attributed to the increased cross‐linking density of GelMA hydrogels. Moreover, the neural stem cells (NSCs) were co‐cultured with the microspheres, and the biocompatibility of the microcarriers was verified by the cell counting kit‐8 (CCK‐8) test (Figure 3D). Statistical analysis was carried out on the first, third, and fifth days, respectively, and the results showed that the neural stem cells proliferated well. The biological activity of the cells was not affected by the microspheres, indicating the good biocompatibility of the microspheres. Therefore, the GelMA microspheres have been proved to be a safe platform for sustained drug release.

### 3.2 Effect of microspheres on NSCs proliferation and differentiation

To study the effect of bFGF‐loaded GelMA microspheres on neural stem cells, the cells were inoculated on a Tissue Culture Polystyrene (TCPS) substrate. Under the proliferation condition, bFGF‐loaded GelMA microspheres or free bFGF were added to the medium, which were set as two experimental groups (bFGF‐GelMA and bFGF group, respectively), while cells co‐cultured with unloaded microspheres (without bFGF) were set as the control group. The cells were cultured in the medium for 3 days, and to evaluate the effect proliferation efficiency, the EdU (5‐ethynyl‐2’‐deoxy‐uridine) proliferation assay was performed. At the same time, the NSCs were stained with Nestin (a marker of neural stem cells) and DAPI (4′,6‐diamidino‐2‐phenylindole and nuclear staining) as shown in Figure S3A. Immunofluorescence images showed that neural stem cells were randomly extended on the standardized substrate, and the cells grew well. The proliferation of cells in the control group was significantly less compared with the experimental groups. Besides, between the two experimental groups, there was no significant difference in promoting the proliferation of neural stem cells (Figure S3B), indicating that bFGF released could effectively promote the proliferation of neural stem cells. This was further confirmed by the Real Time Quantitative Polymerase Chain Reaction (RT‐qPCR) test results (Figure S3C). The expressions of Ki67 and Nestin in the experimental groups were higher than those in the control group, revealing the higher proportion of cells in the proliferation cycle, the faster self‐renewal rate of the neural stem cells, and the stronger stemness. These results demonstrated that the bFGF released from the microspheres could maintain a good bioactivity comparable to fresh bFGF, effectively promoting the proliferation of neural stem cells.

**FIGURE 4:**
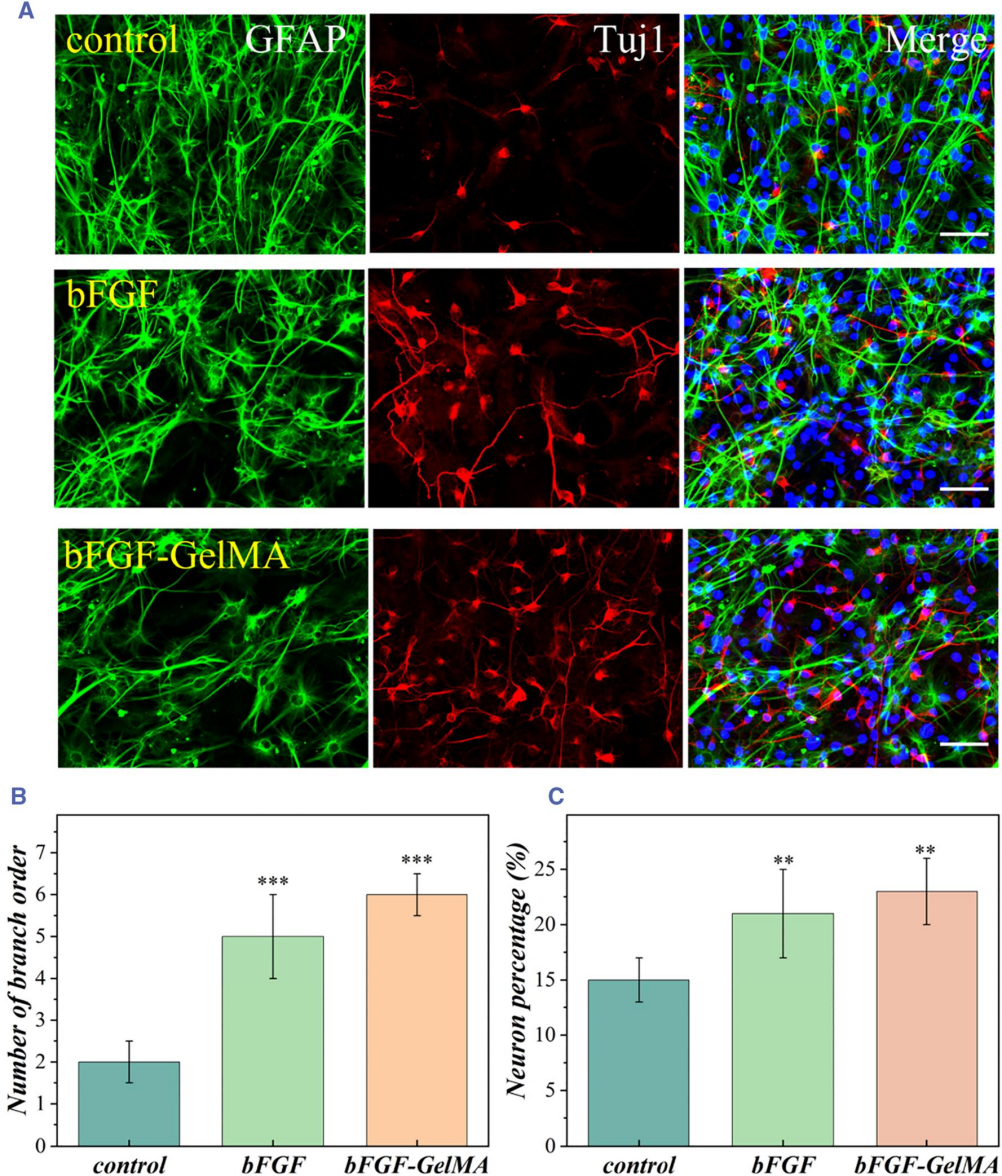
Differentiation of neural stem cells. (A) Fluorescent images of neurons and glial cells cultured under different conditions. Marking Tuj‐1 in red, GFAP in green, and DAPI in blue. (B) Statistics on the number of branching order differentiated by each cell. (C) The differentiation ratio of neural stem cells cultured in different groups. Scale bars are 50 μm.

After the cells were cultured in the proliferation medium for 3 days, they were cultured in the differentiation medium for another 7 days to induce neuronal differentiation. The immunocytochemical method was used to analyze the efficiency of neural differentiation and the growth of neural stem cells in different groups. The cells were stained with the neuronal marker βIII tubulin (Tuj‐1) and the glial marker GFAP.^32^ It was clearly observed that the NSCs successfully differentiate into neurons and glial cells in different groups from the immunofluorescence images (Figure 4A). Then, we measured the number of neuronal branches (the sum of all the dendritic bifurcations of each cell), the differentiation efficiency (the number of differentiated neurons divided by the total number of neural stem cells), and the length of neurites (the sum of all dendritic branches length outside the body of each cell) (Figures 4B,C and S4A). According to the results of immunofluorescence staining, on day 7, the neurons differentiated from neural stem cells developed well in all experimental groups, and a number of neurites extended from the cell body. It was obvious that compared with the control group, the experimental groups showed significantly improved differentiation of neurons and increased number of neuronal branches and length of neurites. Further RT‐qPCR analysis showed that the expression level of Tuj‐1 increased significantly, while the expression level of corresponding GFAP and O4 decreased. This means that most of the NSCs differentiated into neurons and a small number of them differentiated into astrocytes (Figure S4B). Compared with directly adding bFGF, the bFGF‐loaded microspheres with sustained‐release ability were important for the differentiation culture system. This could be ascribed to the fact that the bFGF‐loaded microspheres could continue to supplement bFGF in the culture medium to maintain an effective concentration for cell outgrowth.

### 3.3 Calcium change of NSCs cultured with bFGF‐GelMA microspheres

**FIGURE 5:**
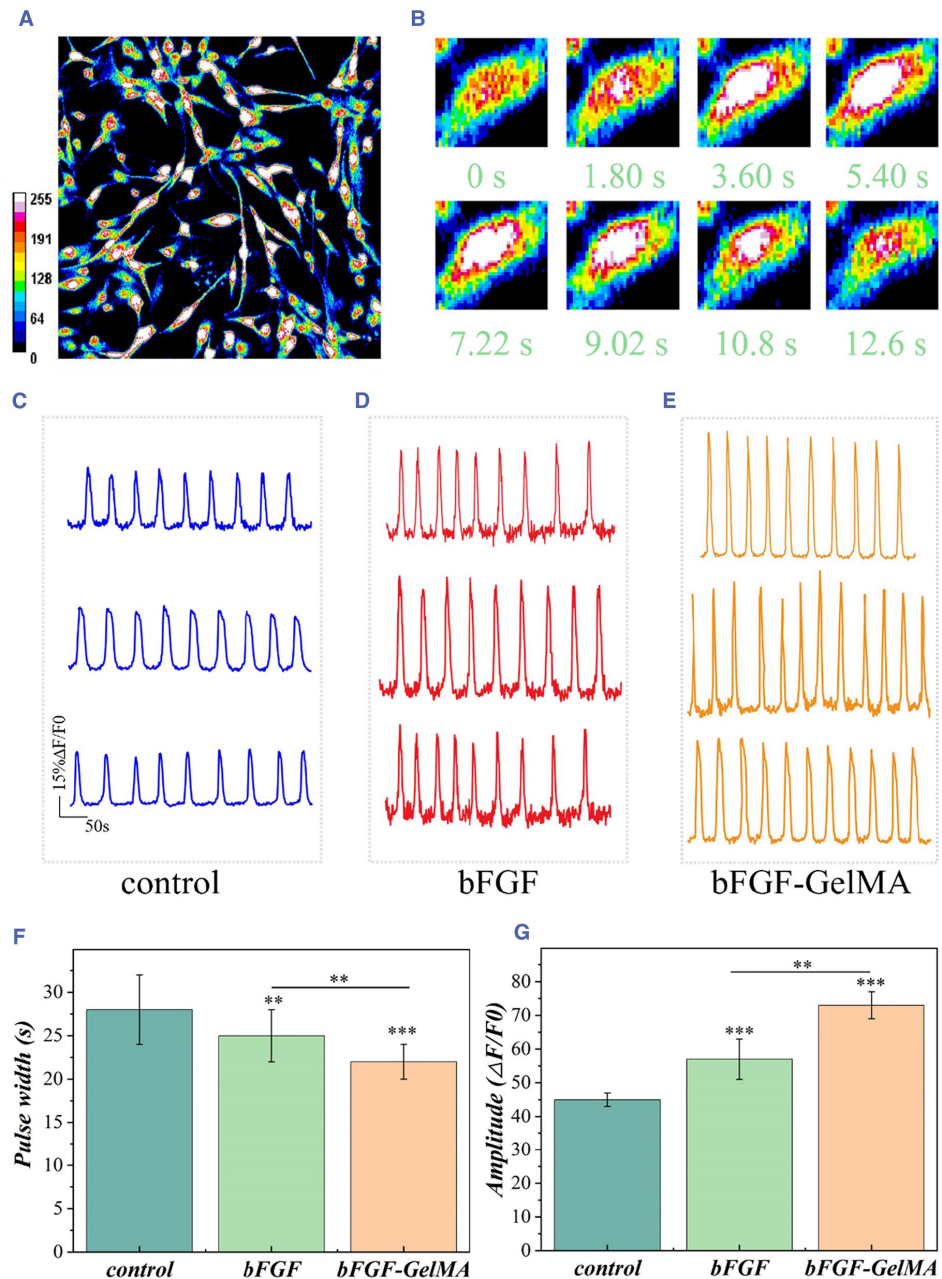
Intracellular Ca^2+^ concentration detected by calcium imaging. (A) Representative fluorescent image of spontaneous Ca^2+^ transients of neural stem cells (NSCs) cultured on the basic fibroblast growth factor‐loaded methacrylate gelatin (bFGF‐GelMA) group. (B) Periodic variation of Ca^2+^ concentration in a single NSC. The recording time is marked, and the colors represent different Ca^2+^ concentrations. (C–E) Representative Ca^2+^ transient traces of NSCs grown in the (C) control group, (D) the bFGF group, and (E) the bFGF‐GelMA group. (F) Statistics of the pulse width after standardized treatment of the intracellular calcium concentration. (G) Statistics of the amplitude of the intracellular calcium concentration after standardized treatment.

Calcium ion (Ca^2+^) is an important signal ion that plays a role in regulating the differentiation of neural stem cells and maturation of neurons.^33^, ^34^ In addition, calcium imaging can also track the action potential of neurons, thus realizing the visualization and quantification of intracellular calcium signals.^35^, ^36^ Using a special fluorescent dye, the concentration of Ca^2+^ in cells is represented by fluorescence intensity to achieve the purpose of detecting neuronal activity.^37^ To determine whether the newborn neurons have functional activity, we conducted fluorescent calcium imaging experiments on neural stem cells. Neural stem cells were inoculated on a TCPS substrate and cultured under different culture conditions for 5 days. Then, fluo‐4 (a calcium indicator dye) was added to incubate for 10 min. The calcium images of the NSCs were collected every 600 ms for a total of 500 cycles. Figure 5A shows the image captured at one moment for cells cultured with bFGF‐loaded GelMA microspheres. To show the calcium transients better, ImageJ is used to convert to a color saturation mode. A series of magnified images were displayed showing the change of the calcium concentration in a single cell during one cycle (Figure 5B). The fluorescence color changed cyclically, which indicated that the intracellular calcium concentration increased at first and then decreased gradually.

We further recorded the flickering brightness and time interval of a single cell and compared the waveforms in each group. We found that the three groups showed sinusoidal waveforms and periodic fluctuations (Figure 5C). To further compare the frequency of the change of the Ca^2+^ concentration, we quantified the pulse width (Figure 5D) and normalized amplitude (Figure 5E) of the waveform after standardized data processing. Generally, the amplitude and pulse width reflected the relative amount of intracellular Ca^2+^ and the duration of Ca^2+^ concussion, respectively. Besides, the frequency is related to the reciprocal of the burst interval. Compared with the control group, the NSCs in the experimental groups showed higher and more synchronous calcium transient frequency and increased amplitude. These results revealed that bFGF could effectively improve the discharge frequency of network activities. Compared with directly adding bFGF, the bFGF‐GelMA group showed a higher peak value of Ca^2+^ and a shorter burst duration of Ca^2+^ spikes, which could be ascribed to the sustained release of bFGF from the GelMA hydrogel microspheres. These results indicated the role of sustained release of bFGF in enhancing the calcium activity of cells as well as patterning of the neuronal network.

### 3.4 In vivo assessment of bFGF‐GelMA microspheres for SCI

**FIGURE 6:**
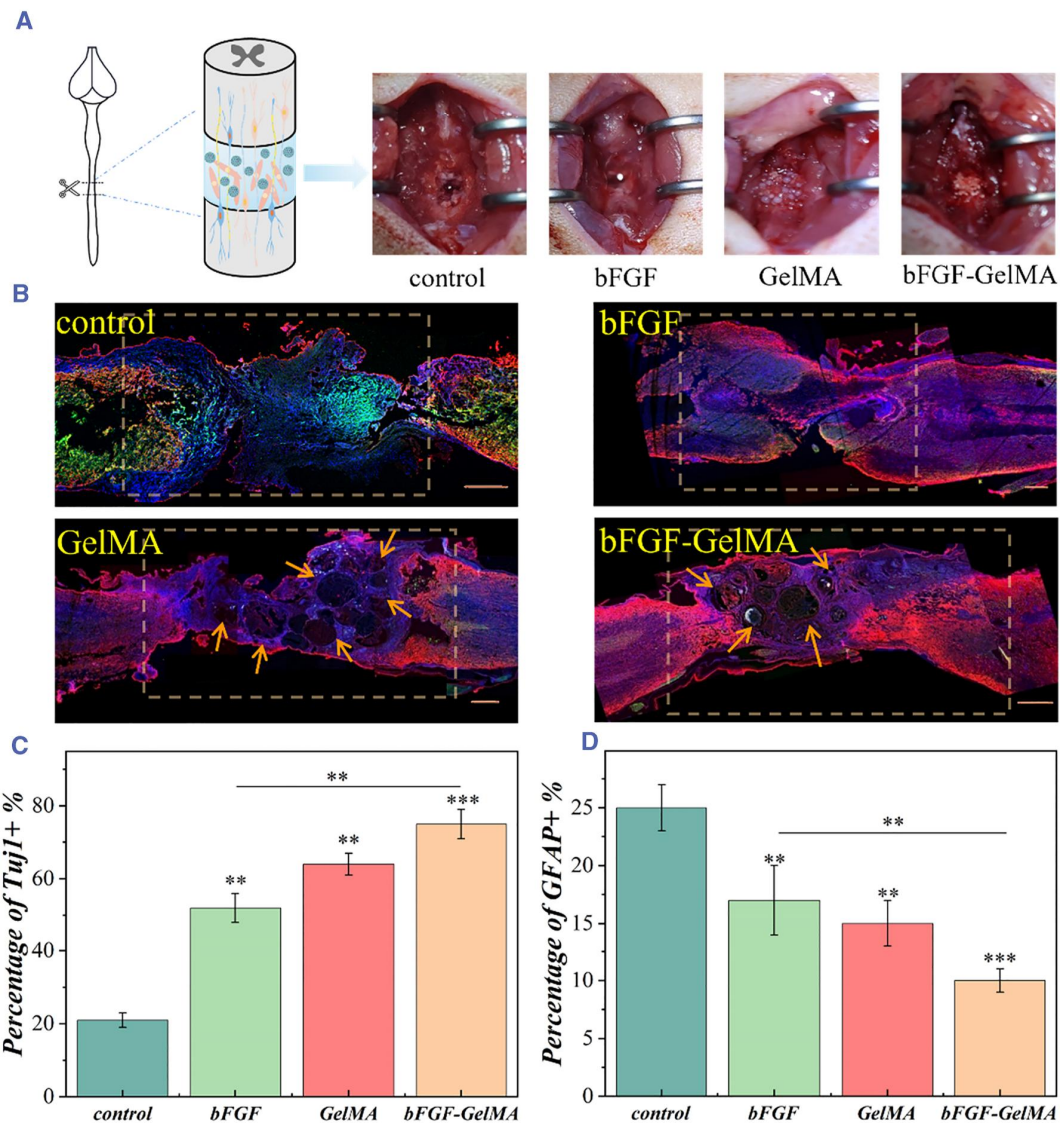
Assessment on the regeneration of the spinal cord tissues. (A) Schematic diagram and the surgical procedures of spinal cord transection with a lesion gap of 2 ± 0.5 mm. (B) Immunofluorescence images of the repaired nerve of different groups (Tuj‐1, red; GFAP, green; DAPI, blue). The arrows point to the position of GelMA microspheres. (C) Quantitative analysis of each group showing the Tuj‐1 positive region. (D) Quantitative analysis of GFAP positive region in each group. Scale bars are 50 μm.

Based on the above results, a rat spinal cord injury model was established to explore the practical application of the bFGF‐loaded hydrogel microspheres in the treatment of SCI (Figure 6A). The rats were then randomly divided into four groups, where they were treated with phosphate buffer saline (PBS, control group), free bFGF (bFGF group), unloaded GelMA microspheres (GelMA group), and bFGF‐load GelMA microspheres (bFGF‐GelMA group) at the injury site. All rats were sacrificed after 8 weeks, and the regenerated tissues at the spinal cord injury site were analyzed longitudinally. Tissue bridging and neuron regeneration were evaluated by immunofluorescence staining and hematoxylin‐eosin (H&E) staining (Figure S5). The neurons were identified by Tuj‐1 and GFAP staining by the immunofluorescence method as shown in Figure 6B. In the control group, the cavity in the injured site was large and the number of neurons was small, while the number of neurons in the bFGF and GelMA groups slightly increased. However, the neuron distribution of these groups was discontinuous, which made it difficult to achieve effective bioelectrical signal transduction, leading to the failure of motor recovery. By contrast, a large number of neurons were observed around the lesion site in the bFGF‐GelMA group, and Tuj‐1 positive neurons were continuously distributed around the microspheres. Such a positive effect induced a bridge between the two segments of the injured spinal cord, which could help to transmit neural activities. Tuj‐1 quantitative analysis showed that the positive staining area of neurons in the lesion site in the bFGF‐GelMA group was 75% ± 4%. It was remarkably higher than that of other groups, specifically speaking, the bFGF group (52% ± 4%), GelMA group (64% ± 3%), and control group (21% ± 2%) (Figure 6C).

The relative distribution of GFAP+ cells is a major maker of glial scar and has been proved to be concerned with the level of nerve tissue regeneration.^38^, ^39^ After treatment, a quantitative analysis for GFAP demonstrated that the positive‐staining area in the bFGF‐GelMA group (10% ± 1%) was memorably lower than that in the bFGF group (17% ± 4%), GelMA group (14% ± 2%), and control group (25% ± 2%) (Figure 6D). In detail, the GFAP positive areas in the SCI group were distinctly higher than in the other groups, which also imply the formation of dense glial scars during repair. By contrast, for the animals treated with the bFGF or GelMA microspheres, the GFAP+ areas were relatively reduced. Furthermore, a superior inhibitory effect of bFGF‐GelMA microspheres for astrocyte overactivity and aggregation was shown by immunofluorescent results. Therefore, the bFGF‐GelMA groups had clearly restrained glial scar formation after SCI (Figure 6B). Additionally, H&E staining results showed different degrees of nerve tissue reconnection between the experimental groups and the blank group (Figure S5). Some tissue junctions were found in the bFGF group, but the intermediate was not connected, while the repaired tissue structure was loose and disordered in the GelMA group. Different from the other groups, the anterior and posterior ends of the tissues in the bFGF‐GelMA group were closely connected with a reduced cavity volume, indicating the outcome of spinal tissue regeneration. These results demonstrated that the bFGF‐loaded GelMA microspheres could promote neuronal differentiation and inhibit the glial scars formation at the lesion site.

Besides, Basso, Beattie and Bresnahan locomotor rating scale (BBB) score was used to analyze the behavior of hind limb movement (Figure S6). We found that the initial BBB score of all spinal cord injury rats was close to 0, followed by varying degrees of recovery. Total spinal cord injury resulted in almost complete paralysis and no motor function in untreated rats (control group) 8 weeks after surgery. In contrast, the hind limb motor function of rats in the other three groups showed a certain degree of recovery. However, compared with the bFGF and GelMA groups, the rats in the bFGF‐GelMA group significantly restored an effective hind leg step rate and a motor function, which resulted in a better BBB score than the other groups at the eighth week. Although the rats did not return to healthy levels, the continued increase in their scores reflected the regeneration of neurons at the site of injury. Moreover, urinary diseases are severe complications of spinal cord injury, which can cause irreversible pathological changes in the bladder and affect the quality of life as well as the overall survival rate of spinal cord injury patients.^40^ Effective remission of urinary tract injury depends on the recovery of spinal nerve function, which is another important indicator of the therapeutic effect.^41^ At 8 weeks after injury, the bladder weight and volume of the rats in the control group and GelMA group increased significantly, while those in the bFGF‐GelMA group basically returned to normal (Figure S7). These results confirmed that the bFGF‐GelMA hydrogel microspheres had a practical protective effect on the urinary system, further supporting their therapeutic role in nerve repair.

## 4 CONCLUSIONS

In summary, we constructed bFGF‐loaded GelMA microspheres exhibiting excellent biocompatibility, degradability, and sustained drug release for spinal cord injury repair. The microspheres were prepared from microfluidic droplets and underwent freeze‐drying to obtain a porous structure. Besides, bFGF could be loaded after appropriate surface modification of the microspheres. Moreover, the prepared GelMA hydrogel microspheres support the controllable release of bFGF, thus promoting the proliferation and differentiation of neural stem cells. Benefiting from these advantages, the bFGF‐loaded microspheres were implanted into the spinal cord injury site of a rat model, finally enhancing nerve regeneration and effectively recovering the hind limb motor function. These results indicated that such drug‐loaded microspheres could provide a novel option for the clinical treatment of spinal cord injury and could become a promising platform for regenerative medicine.

## 5 METHODS

### 5.1 Materials

Methacrylic anhydride and gelatin from Sigma‐Aldrich were purchased, and the concentration of methacrylic anhydride was 94%. Accutase and DMEM/F12 were purchased from Gibco. B27 and kFluor555 Click‐iT EdU were obtained from Invitrogen. bFGF was provided by Wenzhou Medical University. The NeuroCult Differentiation Kit (Mouse) was obtained from Stem Cell. The collagenase type 2 used in the degradation experiment was produced by Worthington. Cell Counting Kit‐8 reagent used in the biocompatibility experiment and the nuclear dye (DAPI) staining solution were Beyotime (Nanjing, China) products. 250 g male Sprague‐Dawley (SD) rats were purchased from Nanjing Qinglongshan Mountain. Animal experiments were treated strictly following the Guide for the Care and Use of Laboratory Animals. All animal schemes received confirmation from the Animal Experimental Ethical Inspection Committee of Southeast University (No. 20210401009).

### 5.2 Preparation of the GelMA microspheres

A microfluidic device was constructed consisting of two cylindrical capillaries arranged coaxially in a square capillary. By introducing the inner and outer fluid phases into the injection tube and collection tube, respectively, a continuous co‐flow system was formed. The outer phase was soybean oil. Different concentrations of the GelMA solution mixed with 1 v/v% photoinitiator were served as the inner phase. When the two phases met at the cross‐junction of the two capillaries, droplets were generated in the collection tube. Finally, the droplets were cross‐linked under UV irradiation and solidified to obtain GelMA hydrogel microspheres. The collected microspheres were washed repeatedly with absolute ethyl alcohol to remove soybean oil. Then, the microspheres were soaked in fresh PBS for 4 h and repeated six times to remove other additives. To obtain porous microspheres, the purified microspheres needed to be frozen and vacuum dried for 48 h.

### 5.3 Preparation of the bFGF‐GelMA microspheres

To construct bFGF‐GelMA microspheres, 100 mg of GelMA microspheres were dispersed in 1 mL of MES (4‐Morpholineethanesulfonic acid) buffer (pH = 6). Then, 8 mg of EDC and 12 mg of NHS were added successively and activated at 37°C for 15 min. Next, 5 μg of bFGF was added and incubated at 37°C for 6 h. The supernatant of bFGF‐GelMA microspheres was obtained by centrifugation and collected for further use.

### 5.4 In vitro drug release study

The release kinetics of FITC‐BSA in the GelMA microspheres was measured in a PBS solution (pH = 7.4). To draw the standard curve of FITC‐BSA, the OD value of FITC‐BSA with different concentrations at 493 nm was detected by a microplate reader (Victor Nivo, PE). The freeze‐dried microspheres 5 mg were weighed into a 5 mL FITC‐BSA solution and oscillated with a constant temperature oscillator for 12 h to fully dissolve. Then, the microspheres were immersed in a 1 mL PBS solution containing collagenase, a 100 μL solution was taken out at different time points for measurement, and then a fresh PBS solution with the same volume was supplemented. The concentration was calculated on the basis of the standard curve. Loading capacity was calculated using the equations:

(1) Loadingcapacity=(massofloadedFITC−BSA/massofmicrospheres)×100%

### 5.5 In vitro degradation behavior of the microspheres

The degradation rate (DR) of the microspheres was measured by immersing the microspheres in a PBS solution, and 2 μg/mL collagenase was added to simulate the degradation environment in vivo. Next, the freeze‐dried samples were weighed at a specified time point. The DR of hydrogel was calculated as follows:

(2) DR=(W0−Wt)/W0×100%

where *W*0 is the dry weight of the initial microspheres and *Wt* represents the weight of the dried sample after degradation.

### 5.6 Extraction, culture, and differentiation of neural stem cells (NSCs)

Neural stem cells were extracted from the hippocampus of fetal mice at 14 days of pregnancy. After being washed with phosphate buffer saline, the hippocampus was digested with accutase. The accutase was then sucked out and digestion was terminated with the addition of PBS. Next, the proliferation medium composed of DMEM/F12, B27 and Epidermal Growth Factor (EGF) was added for cell proliferation. After the passage to the second generation, 1 × 10^5^ cells/well were planted on the laminin‐coated TCPS in 24‐well plates. In vitro cell experiments were divided into three groups, namely the control group (none bFGF culture medium), bFGF group (the concentration of bFGF in the culture medium, 20 ng/mL), and bFGF‐loaded microspheres group (the concentration of bFGF‐loaded microspheres in the culture medium, 300 ng/mL). As to differentiation culture, the medium was NeuroCult Differentiation Kit (Mouse). Importantly, to evaluate the specific effect of the cumulative release of bFGF‐loaded microspheres on the culture of NSCs, we replaced half of the culture medium in each well after 3 days.

### 5.7 Biocompatibility test

The toxicity of the GelMA microspheres to NSCs was detected using the cell counting kit. Briefly, the NSCs were cultured according to the previous protocol. Next, the CCK‐8 solution with the culture medium (1:10) was added to each well and co‐cultured with the cells in a temperature incubator for 2 h. Subsequently, the microplate reader (Victor Nivo, PE) was utilized to detect the OD value at 450 nm of different groups.

### 5.8 EdU labeling

Click‐iT EdU Imaging kit was used for determining the effects of the microspheres on cell proliferation. Briefly, EdU markers were added to the medium and incubated with NSCs after the standard culture of the samples. Subsequently, the Click‐iT reaction buffer was prepared according to the instructions and was incubated with the NSCs at room temperature for 45 min for immunofluorescence staining.

### 5.9 Calcium imaging

The sample was incubated with a standard calcium indicator for 10 min and then cleaned with phenol‐free red DMEM/F12. The images were obtained by a confocal microscope (Zeiss, LSM‐710, Germany) equipped with a water objective. The calcium images were collected every 600 ms for 500 cycles. Finally, ImageJ software (v 1.8.0, National Institute of Mental Health, USA) was used for further analysis.

### 5.10 Animal surgery

Male SD rats (250–300 g) were used to establish the spinal cord model. In detail, rats were removed of the hair on the back near T10 spinous process under deep anesthesia. After the muscle was separated, laminectomy was performed to expose the dorsal surface of T8‐10 segment. Laminectomy was used to fully expose the median vein of the spinal cord. A microscopic shear (14 cm, LEANMED) was used to cut a 2 mm‐long spinal cord, resulting in a nerve defect. Then, a total of 28 rats were divided into the control group, bFGF group (50 μL, 100 μg/mL), GelMA microspheres (without bFGF) group (250 μL, 300 μg/mL), and bFGF‐GelMA microsphere group (250 μL, 300 μg/mL). Animals in the spinal cord injury group were injected with only PBS after injury, and those in the other groups were treated with the corresponding materials to the injured area. After 8 weeks, the spinal cord segments of the injured site were taken for observation.

### 5.11 Immunofluorescence analysis

The cells were first washed with PBS solution, and then fixed with 4% Paraformaldehyde (PFA) at room temperature for 1 h. Next, the cells were washed thrice with 0.1% PBST and blocked in the blocking solution for 1 h. The prepared primary antibodies were incubated overnight at 4°C. After that, cells were incubated with the appropriate secondary antibody and DAPI for 1 h. In the end, the sample was cleaned again with 0.1% PBST, and the DAKO fixed sample was then used for imaging.

### 5.12 Immunohistochemical analysis

The rats were anaesthetized with pentobarbital sodium and sacrificed, PBS was perfused, and the spinal cord segment with a length of 5 mm at the injured site was taken. The spinal cord was fixed with 4% PFA for 2 h at 25°C and stayed overnight at 4°C. Then, they were transferred to sucrose solutions of different concentrations and vacuumed for 1 h. After this, they were transferred to optimal cutting temperature compound (OCT) solutions of different concentrations and vacuumed for 1 h. Finally, the samples were implanted into 100% OCT, embedded, and then sliced longitudinally by a −20°C frozen slicer into 20 μm‐thick sections for H&E staining and immunofluorescence staining.

### 5.13 Statistical analysis

For in vitro tests, three parallel samples were used in all experiments, and all in vivo experiments were repeated seven times. Experimental data were analyzed with Student's test (comparing two groups) and one‐way ANOVA followed by a Dunnett's multiple comparisons (comparing three groups), and all data are presented as the mean ± standard deviation with *p* < 0.05 considered statistically significant (*: *p* < 0.05, * *: *p* < 0.01, * * *: *p* < 0.001).

## AUTHOR CONTRIBUTIONS

Xiaoyan Chen, Lei Ren, Hui Zhang, and Yangnan Hu contributed equally to this work. Renjie Chai, Jian Xiao, Luoran Shang, and Jingwu Sun conceived the idea and designed the experiment. Xiaoyan Chen, Jiaying Cai, Jiamin Guo, and Hong Cheng carried out the experiments and contributed to the scientific discussion of the article. Yingbo Shen, Kaichen Wang, Yanru Qi, and Hao Wei assisted with the experiment operations. Xiaoyan Chen, Hui Zhang, and Lei Ren wrote the manuscript. Menghui Liao and Xiaokun Li performed supervision.

## CONFLICT OF INTEREST STATEMENT

The authors declare that they have no known competing financial interests or personal relationships that could have appeared to influence the work reported in this paper. Luoran Shang is a member of the *Smart Medicine* editorial board.

## ETHICS STATEMENT

All animal experiments were approved by the Animal Experimental Ethical Inspection Committee of Southeast University (No. 20210401009) and were conducted in compliance with the Regulations for the Administration of Affairs Concerning Experimental Animals of China.

## Supporting information

Supplementary Material S1

## References

[1] D. Xu , D. Wu , M. Qin , L. R. Nih , C. Liu , Z. Cao , J. Ren , X. Chen , Z. He , W. Yu , J. Guan , S. Duan , F. Liu , X. Liu , J. Li , D. Harley , B. Xu , L. Hou , I. S. Y. Chen , J. Wen , W. Chen , S. Pourtaheri , Y. Lu , Adv. Mater. 2019, 31, 1900727. 10.1002/adma.201900727 31125138

[2] Z. Álvarez , A. N. Kolberg‐Edelbrock , I. R. Sasselli , J. A. Ortega , R. Qiu , Z. Syrgiannis , P. A. Mirau , F. Chen , S. M. Chin , S. Weigand , E. Kiskinis , S. I. Stupp , Science 2021, 374, 848. 34762454 10.1126/science.abh3602 PMC8723833

[3] S. G. Varadarajan , J. L. Hunyara , N. R. Hamilton , A. L. Kolodkin , A. D. Huberman , Cell 2022, 185, 77. 34995518 10.1016/j.cell.2021.10.029 PMC10896592

[4] T. Wu , J. Zhang , Y. Wang , D. Li , B. Sun , H. El‐Hamshary , M. Yin , X. Mo , Mater. Sci. Eng. C Mater. Biol. Appl. 2018, 82, 121. 29025640 10.1016/j.msec.2017.08.072

[5] C. Piard , A. Jeyaram , Y. Liu , J. Caccamese , S. M. Jay , Y. Chen , J. Fisher , Biomaterials 2019, 222, 119423. 31442885 10.1016/j.biomaterials.2019.119423 PMC6745276

[6] H. Bei , Y. Yang , Q. Zhang , Y. Tian , X. Luo , M. Yang , X. Zhao , Molecules 2019, 24, 658. 30781759 10.3390/molecules24040658 PMC6413135

[7] L. Agrawal , M. Saidani , L. Guillaud , M. Terenzio , Mater. Sci. Eng. C Mater. Biol. Appl. 2021, 131, 112502. 34857288 10.1016/j.msec.2021.112502

[8] G. Li , Q. Han , P. Lu , L. Zhang , Y. Zhang , S. Chen , P. Zhang , L. Zhang , W. Cui , H. Wang , H. Zhang , Research 2020, 2020, 2603048. 32851386 10.34133/2020/2603048 PMC7436332

[9] N. Kong , R. Zhang , G. Wu , X. Sui , J. Wang , N. Y. Kim , S. Blake , D. De , T. Xie , Y. Cao , W. Tao , Proc. Natl. Acad. Sci. U. S. A 2022, 119, e2112696119. 35131941 10.1073/pnas.2112696119 PMC8851555

[10] W. Wang , L. Chang , Y. Shao , D. Yu , J. Parajuli , C. Xu , G. Ying , A. K. Yetisen , Y. Yin , N. Jiang , Eng. Regen. 2022, 3, 1.

[11] Z. Wang , Y. Wang , J. Yan , K. Zhang , F. Lin , L. Xiang , L. Deng , Z. Guan , W. Cui , H. Zhang , Adv. Drug Deliv. Rev. 2021, 174, 504. 33991588 10.1016/j.addr.2021.05.007

[12] X. Huang , G. Wu , C. Liu , X. Hua , Z. Tang , Y. Xiao , W. Chen , J. Zhou , N. Kong , P. Huang , J. Shi , W. Tao , Nano Lett. 2021, 21, 9706. 34723546 10.1021/acs.nanolett.1c03539

[13] C. Li , Y. Liu , M. Wei , J. Liu , X. Yu , P. Hu , Y. Liu , Eng. Regen. 2022, 3, 73.

[14] H. Liu , Z. Cai , F. Wang , L. Hong , L. Deng , J. Zhong , Z. Wang , W. Cui , Adv. Sci. 2021, 8, 2101619. 10.1002/advs.202101619 PMC8456273 34292669

[15] D. Huang , X. Zhang , X. Fu , Y. Zu , W. Sun , Y. Zhao , Eng. Regen. 2021, 2, 246.

[16] D. Wei , R. Qiao , J. Dao , J. Su , C. Jiang , X. Wang , M. Gao , J. Zhong , Small 2018, 14, 1800063. 10.1002/smll.201800063 29682876

[17] L. Wu , Y. Gu , L. Liu , J. Tang , J. Mao , K. Xi , Z. Jiang , Y. Zhou , Y. Xu , L. Deng , L. Chen , W. Cui , Biomaterials 2020, 227, 119555. 31655445 10.1016/j.biomaterials.2019.119555

[18] J. Bian , F. Cai , H. Chen , Z. Tang , K. Xi , J. Tang , L. Wu , Y. Xu , L. Deng , Y. Gu , W. Cui , L. Chen , Nano Lett. 2021, 21, 2690. 33543616 10.1021/acs.nanolett.0c04713

[19] D. H. Kim , J. Huegel , B. L. Taylor , C. A. Nuss , S. N. Weiss , L. J. Soslowsky , R. L. Mauck , A. F. Kuntz , Acta Biomater. 2020, 111, 341. 32428684 10.1016/j.actbio.2020.04.048 PMC7868956

[20] B. Wu , C. Yang , B. Li , L. Feng , M. Hai , C. Zhao , D. Chen , K. Liu , D. A. Weitz , Small 2020, 16, 2002716. 10.1002/smll.202002716 32578400

[21] J. Zhang , Y. Li , J. Xiong , H. Xu , G. Xiang , M. Fan , K. Zhou , Y. Lin , X. Chen , L. Xie , H. Zhang , J. Wang , J. Xiao , Bioact. Mater. 2021, 6, 3177. 33778197 10.1016/j.bioactmat.2021.03.001 PMC7970014

[22] L. Chen , Y. Xiao , Q. Wu , X. Yan , P. Zhao , J. Ruan , J. Shan , D. Chen , D. A. Weitz , F. Ye , Small 2021, 17, 2102579. 10.1002/smll.202102579 34390183

[23] W. Zhuge , H. Liu , W. Wang , J. Wang , Eng. Regen. 2022, 3, 110.

[24] T. Xin , J. Mao , L. Liu , J. Tang , L. Wu , X. Yu , Y. Gu , W. Cui , L. Chen , ACS Appl. Mater. Interfaces 2020, 12, 6840. 31999085 10.1021/acsami.9b18496

[25] W. Li , J. Chen , S. Zhao , T. Huang , H. Ying , C. Trujillo , G. Molinaro , Z. Zhou , T. Jiang , W. Liu , L. Li , Y. Bai , P. Quan , Y. Ding , J. Hirvonen , G. Yin , H. A. Santos , J. Fan , D. Liu , Nat. Commun. 2022, 13, 1262. 35273148 10.1038/s41467-022-28787-7 PMC8913677

[26] Y. Yang , T. Xu , Q. Zhang , Y. Piao , H. Bei , X. Zhao , Small 2021, 17, 2006598. 10.1002/smll.202006598 33705605

[27] Y. Piao , H. You , T. Xu , H. Bei , I. Z. Piwko , Y. Y. Kwan , X. Zhao , Eng. Regen. 2021, 2, 47.

[28] A. C. Daly , L. Riley , T. Segura , J. A. Burdick , Nat. Rev. Mater. 2020, 5, 20. 34123409 10.1038/s41578-019-0148-6 PMC8191408

[29] R. Li , Y. Li , Y. Wu , Y. Zhao , H. Chen , Y. Yuan , K. Xu , H. Zhang , Y. Lu , J. Wang , X. Li , X. Jia , J. Xiao , Biomaterials 2018, 168, 24. 29609091 10.1016/j.biomaterials.2018.03.044 PMC5935004

[30] Y. Zhou , Z. Wang , J. Li , X. Li , J. Xiao , J. Cell Mol. Med. 2018, 22, 25. 29063730 10.1111/jcmm.13353 PMC5742738

[31] S. Zhu , Y. Ying , J. Ye , M. Chen , Q. Wu , H. Dou , W. Ni , H. Xu , J. Xu , Cell Death Dis. 2021, 12, 274. 33723238 10.1038/s41419-021-03546-6 PMC7960741

[32] W. Ji , Z. Álvarez , A. N. Edelbrock , K. Sato , S. I. Stupp , ACS Appl. Mater. Interfaces 2018, 10, 41046. 30475573 10.1021/acsami.8b13653

[33] A. B. Toth , A. K. Shum , M. Prakriya , Cell Calcium 2016, 59, 124. 27020657 10.1016/j.ceca.2016.02.011 PMC5228525

[34] A. Gengatharan , S. Malvaut , A. Marymonchyk , M. Ghareghani , M. Snapyan , J. Fischer‐Sternjak , J. Ninkovic , M. Götz , A. Saghatelyan , Cell 2021, 184, 709. 33482084 10.1016/j.cell.2020.12.026

[35] Y. Bando , M. Wenzel , R. Yuste , Nat. Commun. 2021, 12, 7229. 34893595 10.1038/s41467-021-27444-9 PMC8664861

[36] R. A. de Melo Reis , H. R. Freitas , F. G. de Mello , Front. Neurosci. 2020, 14, 569361. 33122991 10.3389/fnins.2020.569361 PMC7566175

[37] H. Dana , Y. Sun , B. Mohar , B. K. Hulse , A. M. Kerlin , J. P. Hasseman , G. Tsegaye , A. Tsang , A. Wong , R. Patel , J. J. Macklin , Y. Chen , A. Konnerth , V. Jayaraman , L. L. Looger , E. R. Schreiter , K. Svoboda , D. S. Kim , Nat. Methods 2019, 16, 649. 31209382 10.1038/s41592-019-0435-6

[38] X. Liu , M. Hao , Z. Chen , T. Zhang , J. Huang , J. Dai , Z. Zhang , Biomaterials 2021, 272, 120771. 33798962 10.1016/j.biomaterials.2021.120771

[39] L. Li , Y. Zhang , J. Mu , J. Chen , C. Zhang , H. Cao , J. Gao , Nano Lett. 2020, 20, 4298. 32379461 10.1021/acs.nanolett.0c00929

[40] S. A. Quadri , M. Farooqui , A. Ikram , A. Zafar , M. A. Khan , S. S. Suriya , C. F. Claus , B. Fiani , M. Rahman , A. Ramachandran , I. I. T. Armstrong , M. A. Taqi , M. M. Mortazavi , Neurosurg. Rev. 2020, 43, 425. 29998371 10.1007/s10143-018-1008-3

[41] J. J. Wyndaele , Nat. Rev. Urol. 2016, 13, 705. 27779229 10.1038/nrurol.2016.206

