# Supplementary material for: Basic fibroblast growth factor‐loaded methacrylate gelatin hydrogel microspheres for spinal nerve regeneration
Source: Smart Med. 2023 Mar 28;2(2):e20220038. doi: 10.1002/SMMD.20220038 (PMC11235853; doi:10.1002/SMMD.20220038)
Supplement: Supplementary file 1 — Supplementary Material S1 [file SMMD-2-e20220038-s001.docx]

**Supplementary**

**Basic fibroblast growth factor‐loaded methacrylate gelatin hydrogel microspheres for spinal nerve regeneration**

Xiaoyan Chen^1^, Lei Ren^1^, Hui Zhang^1^, Yangnan Hu^1^, Menghui Liao^1^, Yingbo Shen^2^, Kaichen Wang^2^, Jiaying Cai^1^, Hong Cheng^1^, Jiamin Guo^1^, Yanru Qi^1^, Hao Wei^3^, Xiaokun Li^4,5^, Luoran Shang^6,*^, Jian Xiao^4,5,7,*^, Jingwu Sun^8,*^, Renjie Chai^1,2,4,9,10,11,*^

^1^State Key Laboratory of Bioelectronics, Department of Otolaryngology Head and Neck Surgery, Zhongda Hospital, School of Life Sciences and Technology, Advanced Institute for Life and Health, Jiangsu Province High Tech Key Laboratory for Bio-Medical Research, Southeast University, Nanjing 210096, China

^2^Chien-Shiung Wu College, Southeast University, Nanjing 210096, China

^3^Department of Otolaryngology Head and Neck Surgery, Affiliated Drum Tower Hospital of Nanjing University Medical School, Nanjing 210008, China

^4^Co-Innovation Center of Neuroregeneration, Nantong University, Nantong 226001, China

^5^School of Pharmaceutical Sciences, Wenzhou Medical University, Wenzhou, Zhejiang 325035, China

^6^Shanghai Xuhui Central Hospital, Zhongshan-Xuhui Hospital, and the Shanghai Key Laboratory of Medical Epigenetics, the International Co-laboratory of Medical Epigenetics and Metabolism (Ministry of Science and Technology), Institutes of Biomedical Sciences, Fudan University, Shanghai, China

^7^Oujiang Laboratory (Zhejiang Lab for Regenerative Medicine, Vision and Brain Health), Wenzhou, Zhejiang 325001, China]

^8^Department of Otolaryngology-Head and Neck Surgery, The First Affiliated Hospital of USTC, Division of Life Sciences and Medicine, University of Science and Technology of China, Hefei, Anhui, 230001, China

^9^Department of Otolaryngology Head and Neck Surgery, Sichuan Provincial People's Hospital, University of Electronic Science and Technology of China, Chengdu 610072, China

^10^Institute for Stem Cell and Regeneration, Chinese Academy of Science, Beijing 100086, China

^11^Beijing Key Laboratory of Neural Regeneration and Repair, Capital Medical University, Beijing 100069, China


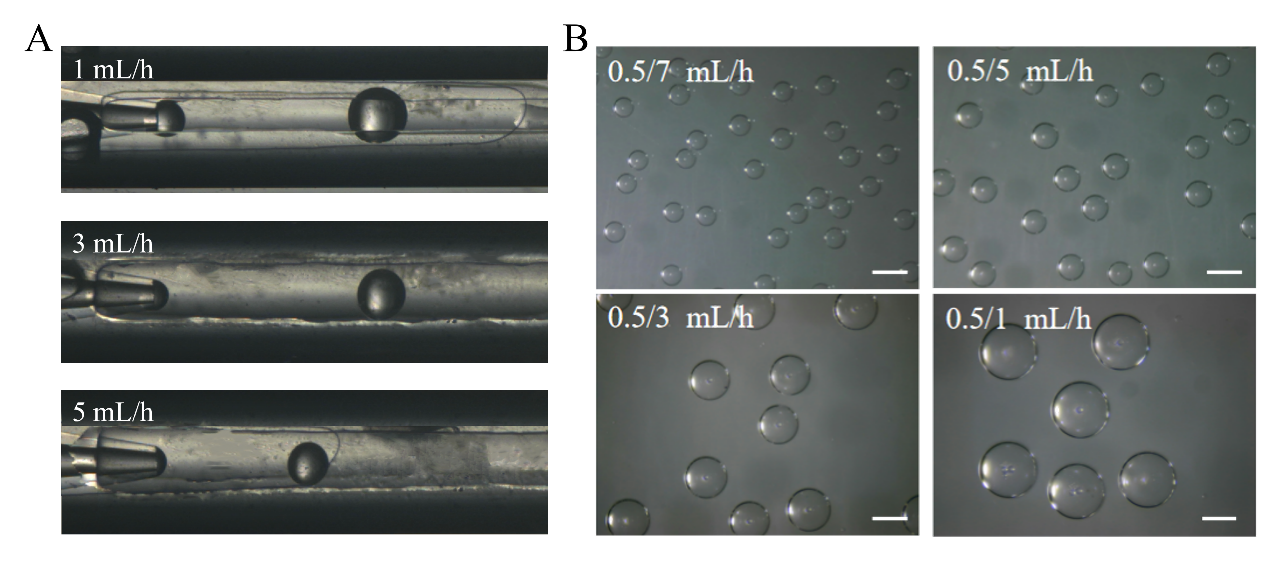


**Figure S1**. (A) Digital photographs of the generation of GelMA pre-gel droplets in the microfluidic channel with varying outer flow rates. The inner flow rate was fixed at 0.5 mL/h. (B) Microscopic images of the GelMA microspheres fabricated at different flow rates. Scale bars are 250 µm.


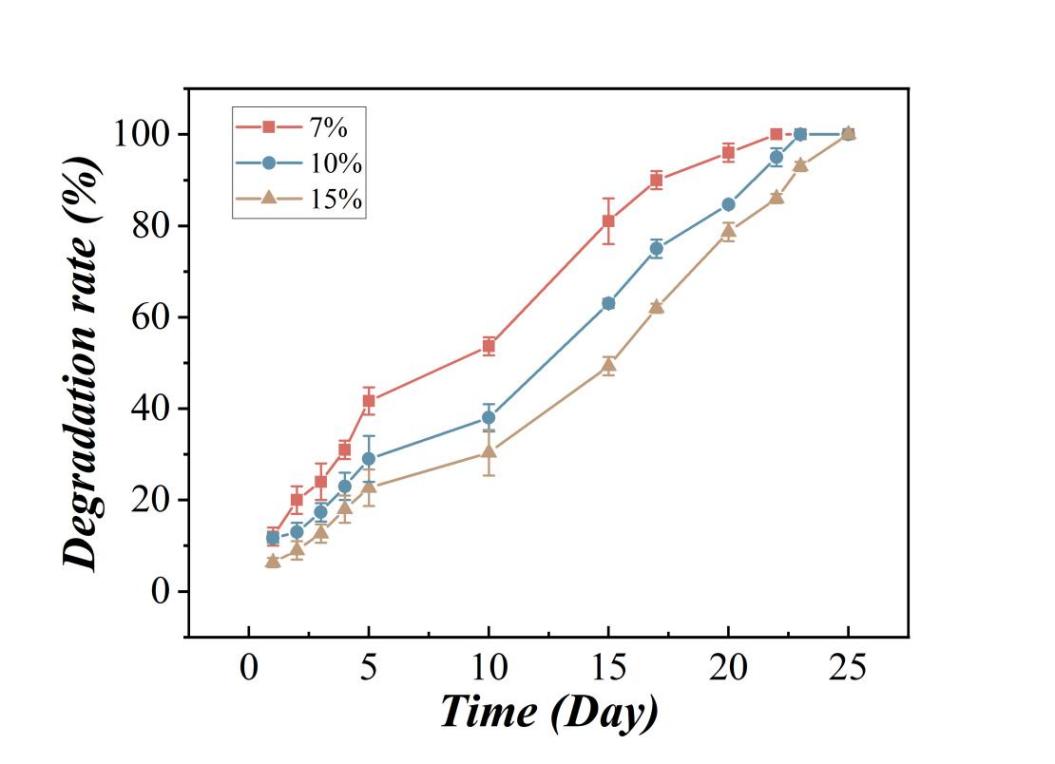


**Figure S2.** The degradation rate of GelMA hydrogel microspheres with different GelMA concentrations.


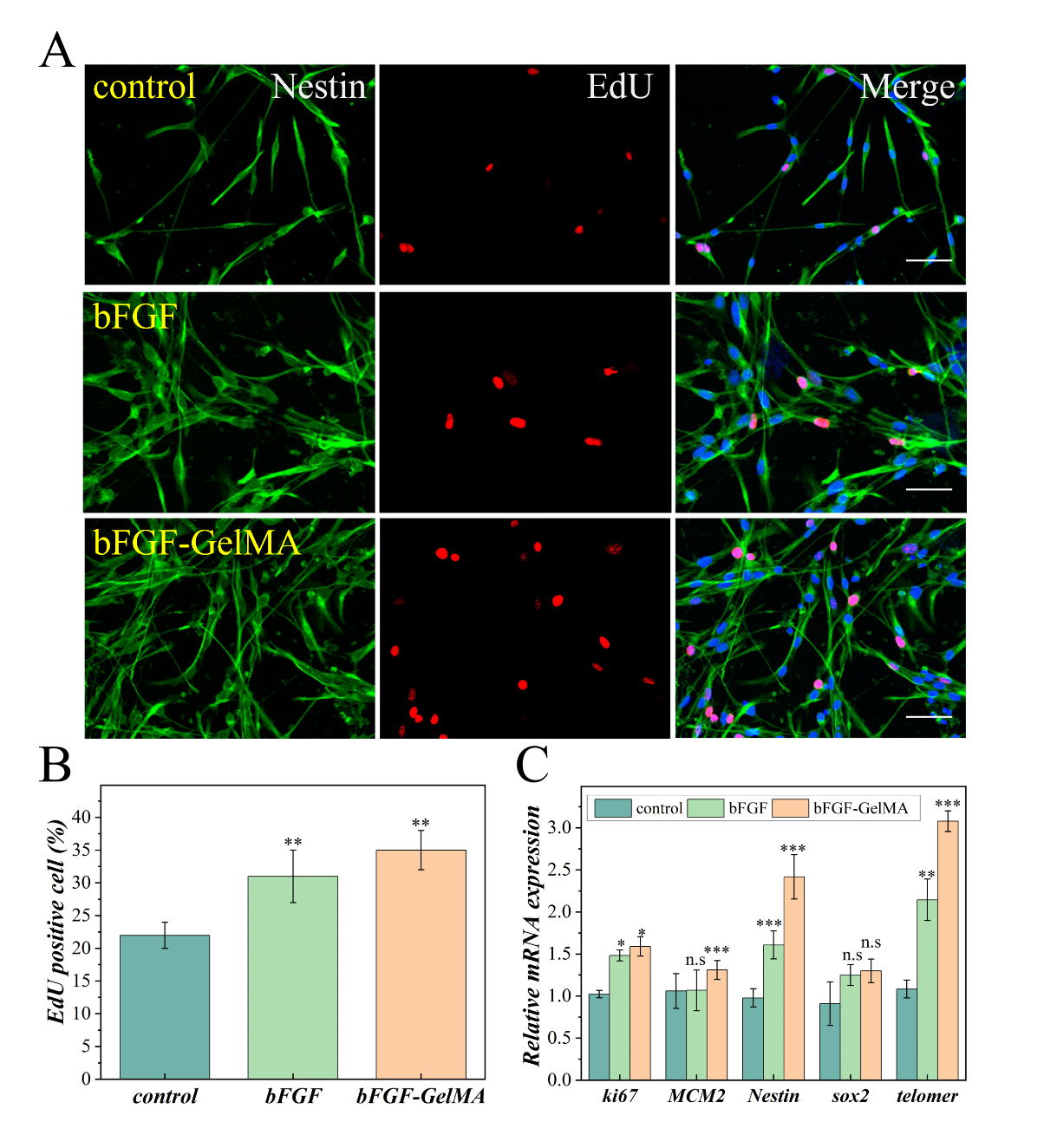


**Figure S3.** Effects of bFGF-loaded GelMA microspheres on the proliferation of NSCs. (A) Representative fluorescent images of NSCs stained with Nestin (in green), EdU (in red), and DAPI (in blue). (B) Histogram of the EdU-positive cell percentage. (C) The expression of proliferation-related genes in NSCs determined by RT-qPCR. Scale bars are 50 µm.


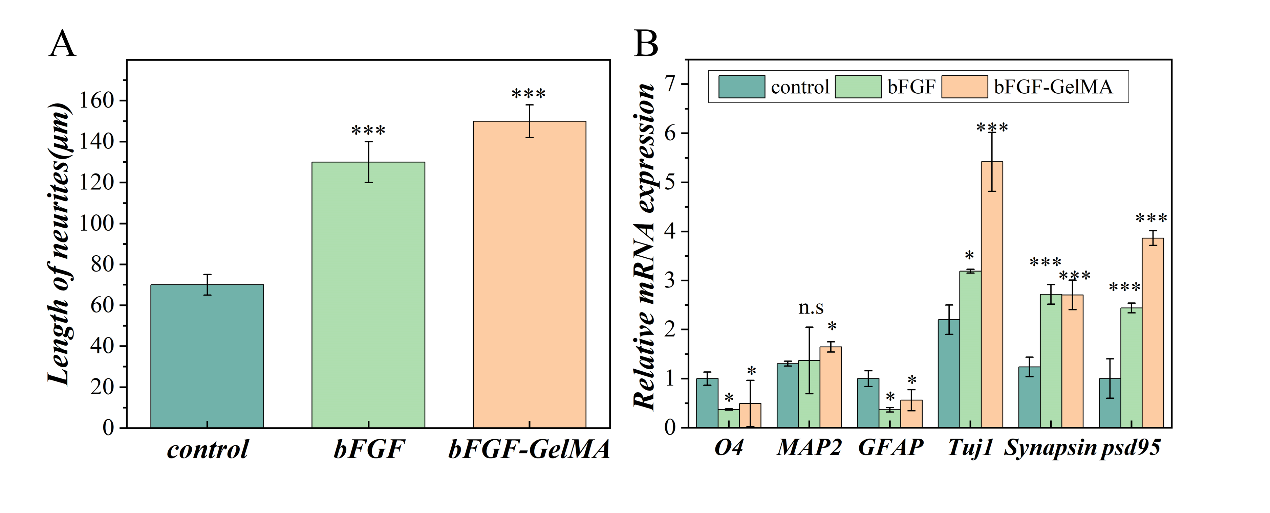


**Figure S4.** (A) The length of neurites (the sum of all dendritic branches outside the body of each cell) in each group. (B) Relative mRNA expression of cells in each group.


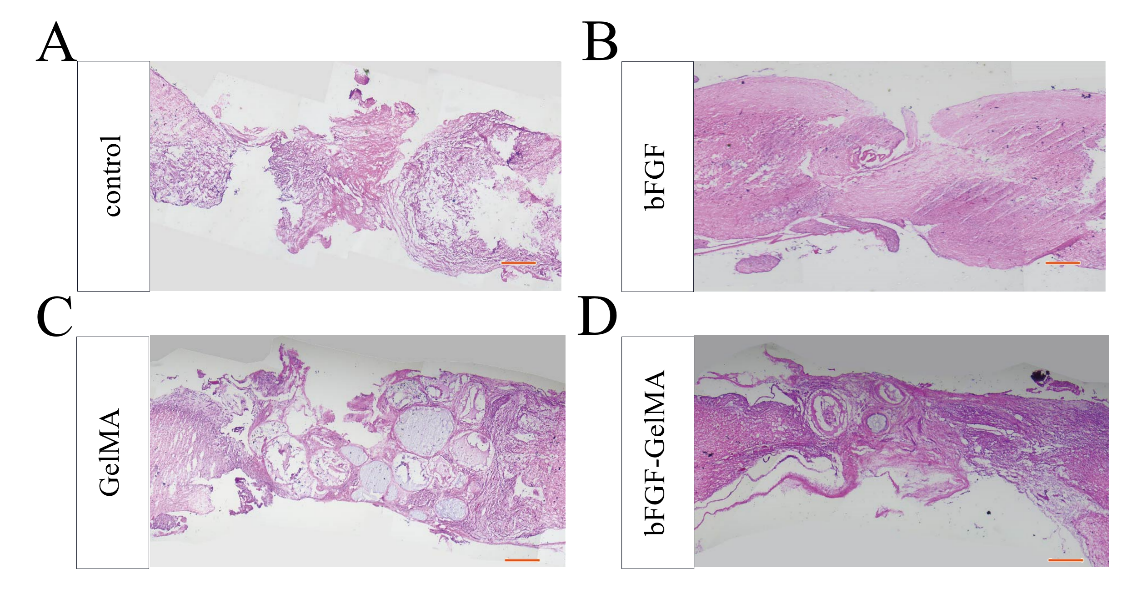


**Figure S5**. H&E staining of nerve repair of different groups. Scale bars are 500 µm.


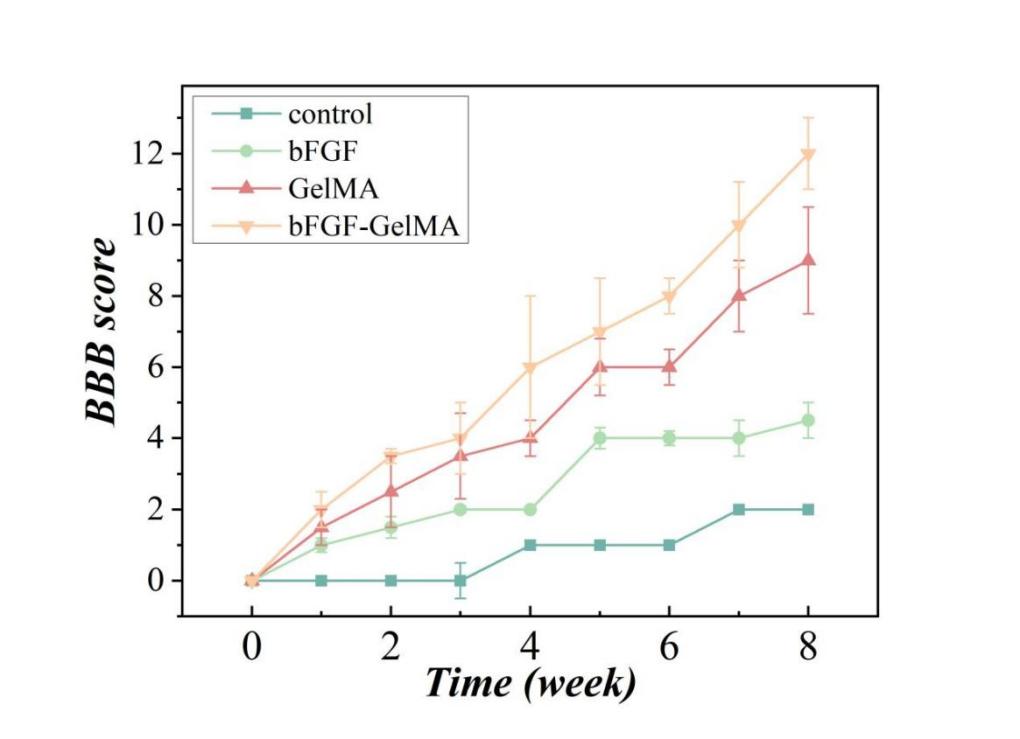


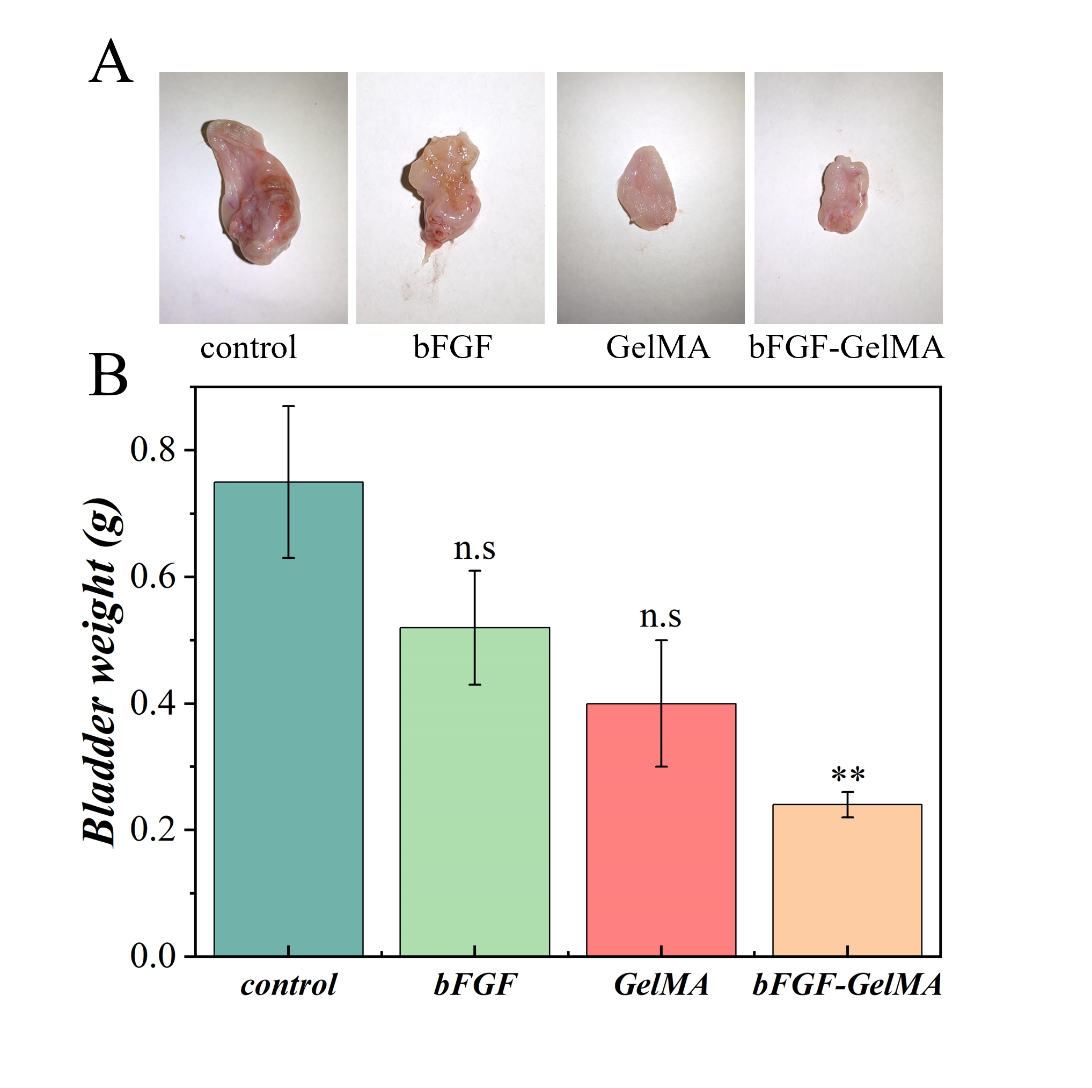
**Figure S6**. Animal BBB scores during 8 weeks of treatment.

**Figure S7**. (A) Photographs of bladders of rats in different groups. (B) Animal bladder weight during 8 weeks of treatment.
